# Potential urinary volatile organic compounds as screening markers in cancer – a review

**DOI:** 10.3389/fonc.2024.1448760

**Published:** 2024-11-25

**Authors:** Alexandre Goertzen, Biniam Kidane, Naseer Ahmed, Michel Aliani

**Affiliations:** ^1^ Division of Neurodegenerative Disorders, Saint Boniface Hospital Albrechtsen Research Center, Winnipeg, MB, Canada; ^2^ Department of Food and Human Nutritional Sciences, University of Manitoba, Winnipeg, MB, Canada; ^3^ Paul Albrechtsen Research Institute, CancerCare, Winnipeg, MB, Canada; ^4^ Department of Surgery, College of Medicine, University of Manitoba, Winnipeg, MB, Canada; ^5^ Rady Faculty of Health Sciences, Department of Radiology, Section of Radiation Oncology, Winnipeg, MB, Canada; ^6^ Department of Physiology and Pathophysiology, University of Manitoba, Winnipeg, MB, Canada

**Keywords:** cancer, biomarkers, volatile organic compounds, gas chromatography mass spectrometry, electronic nose, diagnostics, early detection, animal olfaction

## Abstract

Early detection of cancer typically facilitates improved patient outcomes; however, many cancers are not easily diagnosed at an early stage. One potential route for developing new, non-invasive methods of cancer detection is by testing for cancer-related volatile organic compounds (VOCs) biomarkers in patients’ urine. In this review, 44 studies covering the use and/or identification of cancer-related VOCs were examined, including studies which examined multiple types of cancer simultaneously, as well as diverse study designs. Among these studies the most studied cancers included prostate cancer (29% of papers), lung cancer (22%), breast cancer (20%), and bladder cancer (18%), with a smaller number of studies focused on colorectal cancer, cervical cancer, skin, liver cancer and others. Importantly, most studies which produced a VOC-based model of cancer detection observed a combined sensitivity and specificity above 150%, indicating that urine-based methods of cancer detection show considerable promise as a diagnostic tool. Mass spectrometry (MS) and electronic noses (eNose) were the most employed tools used in the detection of VOCs, while animal-based models were less common. In terms of VOCs of interest, 47 chemical species identified as correlated with various types of cancer in at least two unrelated papers, some of which were consistently up- or down-regulated in cancer patients, and which may represent useful targets for future studies investing urinary VOC biomarkers of cancer. Overall, it was concluded that research in this field has shown promising results, but more work may be needed before the widespread adoption of these techniques takes place.

## 1 Introduction

Early detection of cancer will significantly increase the probability of successful treatment, while late detection often results in advanced diseases at presentation and more limited treatment options (1). Cancer is a leading cause of death in Canada, and worldwide, warranting the development of new tools and techniques to facilitate early cancer detection, which could provide substantive benefits to both patients, as well as to healthcare providers (2, 3). These new detection methods would be particularly valuable for some types of cancer which are either difficult to detect, such as pancreatic ductal adenocarcinoma, hepatocellular carcinoma, colorectal cancer, and lung cancer; or are otherwise significantly deadlier if not detected early, such as breast cancer, prostate cancer, or skin cancer (**Table 1**) (3, 4).

**Table 1:** Five-year mortality rates of various cancers at early or late stage – seer cancer statistics.

Location of Cancer	Five-Year Morality Rates		Reference
	Early stage/ Localized	Late stage/Distant	
Pancreas	42.2%	3.1%	(4)
Liver and Intrahepatic Bile Duct	38.1%	3.9%	(4)
Colon & Rectum	91.9%	17.2%	(4)
Urinary Bladder (Invasive & *In Situ*)	65.2%	8.0%	(4)
Lung and Bronchus	68.7%	10.6%	(4)
Breast cancer	99.6%	31.9%	(4)
Prostate cancer	100.0%	36.6%	(4)
Skin Cancer (Melanoma)	100.0%	37.2%	(4)

One potential route for developing these new methods is by creating diagnostic tests for the presence or concentrations of specific compounds, or biomarkers, which can be used as proxies for metabolic processes occurring within the body. Depending on the compounds chosen, this strategy may allow healthcare providers to identify subtle physiological changes in an individual brought on by a particular disease condition, potentially before the onset of any outward symptoms (5). Many different types of compounds have already been extensively used as biomarkers for cancer detection, including proteins (prostate-specific antigen, carcinoembryonic antigen, carbohydrate antigen 19-9, etc.), and nucleic acids/genes (BRCA1, BRCA2, and KRAS genes). However, some of these biomarker tests require relatively complicated and specialized methods to obtain and extract, or require samples be taken from blood or other tissues – which is often highly invasive and requires specialized medical expertise (5).

Smaller volatile organic compounds (VOCs), offer the potential to be more convenient as biomarkers due to their characteristic ability to spontaneously evaporate into a gaseous state, separating themselves from a particular sample without the need for extraction procedures. These compounds are naturally exhaled in breath and excreted in urine and are usually thought to originate as the end products of certain metabolic processes. As a result, monitoring the presence or concentration of these compounds can be used to identify metabolic changes brought on by disease conditions, including cancer (6, 7). Notably, breath and urine are often considered to be most convenient types of samples when developing a diagnostic test, as these fluids are naturally and routinely expelled from the body and can be simply be collected thereafter (8).

While this review focuses on urine-based testing exclusively, it is important to acknowledge that numerous studies have explored breath-cased cancer detection systems, including some included in this review itself which sought to compare the two sample types, and moreover that such methods do offer several advantages (8–11). For instance, breath testing is extremely simple and almost entirely non-invasive – requiring only that patients exhale into a specialized device, something that can be done multiple times per day and on demand. In comparison, collecting urine samples is not necessarily possible at times, as certain health conditions can make urinating on demand difficult, and urine sampling can be perceived as awkward or embarrassing for patients (12).

On the other hand, urine sampling does offer its own advantages. For instance, urine is a much more voluminous and concentrated fluid compared to a breath sample, and therefore may contain more and a higher concentration of VOCs of interest (11). Urine collection also requires very few components as patients must simply urinate into a special sealable cup, and samples can thereafter be frozen for storage. Breath sampling in turn generally requires the use of specialized devices which serve the purpose of preserving and concentrating the sample prior to subsequent analyses (12). Finally, it should be noted that both breath and urine sample composition can be significantly altered by various non-disease factors, and which may need be corrected for when developing a diagnostic tool. In the case of breath sampling, these include factors such as breathing patterns, lung volume, diet, smoking, or environmental exposure (11, 13); while for urination notable factors include diet and level of hydration, time of urination, the presence of medications or supplements, levels of physical activity and others (5, 11, 14–16).

## 2 Discussion

The purpose of this review was to provide a general summary of published literature in the last eleven years, and particularly within the last five years, related to the use of urinary VOCs as potential biomarkers for various types of cancer. To achieve this, published articles were searched using keywords. cancer, volatile’, and ‘urine’. From this initial search, a total of 711 articles were initially identified, and then manually screened for suitability of inclusion. Articles which did not directly relate to the identification, measurement, or at least recognition of volatile compounds, or patterns thereof, produced from the urine of cancer patients were excluded. This left 44 papers to be included in the final review, specific VOCs identified in these papers were included only if they were present exclusively in the cancer patient group, or else were described as statistically significantly up- or down-regulated in cancer patients by the authors. A list of all the referenced papers is included in **Table 2**.

**Table 2:** List of studies related to urinary cancer vocs published over the last eleven years.

Cancer Type	Level of Analysis	Ref
2023		
Prostate cancer	Compared cancer stages I-III, and stages IV & V.	(47)
Lung cancer	Compared patient survival times (3 grades).	(53)
Head and neck cancer, gastrointestinal cancer		(11)
Kidney cancer, gastric cancer, lung cancer	Compared different cancer types.	(60)
Lung cancer		(23)
Breast cancer		(14)
Prostate cancer		(58)
Breast cancer		(48)
Lung cancer	Compared 4 stages of lung cancer, as well as different lung cancer types	(61)
Prostate cancer		(22)
Kidney cancer	eNose	(9)
Esophageal cancer	Screening before and after preoperative chemotherapy	(62)
2022		
Bladder cancer		(42)
Breast cancer	Monitored cancer treatment progression (mice model).	(49)
Bladder cancer	Compared recurrent cancer and non-recurrent patients.	(45)
Bladder cancer	Compared grade and prognosis.	(38)
Breast cancer		(24)
Lymphoma, melanoma, hemangiosarcoma		(63)
2021		
Pancreatic cancer	Compared pancreatic ductal adenocarcinoma and pancreatitis.	(27)
Prostate cancer and bladder cancer		(57)
Breast cancer	Compared mice injected with tumors over time.	(15)
Bladder cancer	Compared grades of cancer	(51)
Cervical cancer	Compared subtypes of cervical cancer	(48)
Prostate cancer		(16)
Prostate cancer	Compared benign and aggressive cancer	(28)
Prostate cancer	Aggressive cancers	(29)
Lung cancer		(8)
Breast cancer	Compared 6 stages and different subtypes of breast cancer	(20)
2020		
Prostate cancer, bladder cancer, kidney cancer	Compared different types of cancer	(43)
Heptatocellcular carcinoma, prostate cancer, bladder cancer		(21)
2019		
Lung cancer	Compared before and after treatment	(31)
Colorectal cancer, adenoma	Compared cancer and adenomas	(64)
Prostate cancer		(50)
Prostate cancer		(41)
Breast cancer/ bone metastasis	Compared cancer induced in mice and human cell lines	(65)
Pancreatic cancer	Compared pancreatic ductal adenocarcinoma and pancreatitis	(18)
Bladder cancer		(37)
2018-2012		
Pancreatic cancer	Compared cancer stages (I & II) and (III & IV)	(19)
Lung cancer	Compared small cell, non-small cell, squamous cell LCs	(66)
Lung cancer		(59)
Prostate cancer		(46)
Lung cancer	Compared non-small cell and small cell lung cancer	(10)
Prostate cancer	Compared stages of cancer and after receiving treatment	(67)
Lung cancer	Compared lung cancer types	(55)

### 2.1 Types of cancer associated with specific VOCs

The studies examined in this review were found to have mainly focused on prostate cancer (covered by 29% of articles), lung cancer (22%), breast cancer (20%), and bladder cancer (18%), while fewer studies were dedicated to pancreatic ductal adenocarcinoma, renal cell adenocarcinoma, and other cancers (4%). Note that in several studies, multiple cancer types were analyzed together. This distribution is justifiable, given prostate cancer, bladder cancer, and breast cancer, all have relatively high 5-year survival rates when detected at an early stage, and as such any new methods which can increase their rate of early detection should be considered highly desirable. Moreover, studies focused on lung cancer, breast cancer, and prostate cancer can further be justified due to these cancers being responsible for the 1^st^, 4^th^, and 5^th^ most US cancer-related deaths between 2018 to 2022, and as such represent some of the most lethal cancers overall. On the other hand, colorectal cancer, and pancreatic ductal adenocarcinoma, despite being responsible for the 2^nd^ and 3^rd^ most US cancer deaths between 2018 to 2022, appear to have received considerably less attention from researchers (4). In the former case, this apparent disinterest may be because several new diagnostic technologies are already under development, including methods based on DNA analysis from fecal samples, monitoring changes in the gut microbiome, and using the detection of VOCs present in breath (17). Pancreatic ductal adenocarcinoma, in turn, may be relatively overlooked due to a combination of being relatively rare, and highly lethal even when detected at earlier stages, making it a less attractive option for developing new diagnostic tools (18). Alternatively, it may be difficult to separate the effects of pancreatic ductal adenocarcinoma from pancreatitis, possibly reducing the potential value of a VOC-based diagnostic tool for this specific disease (19).

### 2.2 Sample selection strategies

Unsurprisingly, a diverse set of sampling strategies were employed by the various studies included in this review, the details of which are summarized in **Table 3**. Despite this, several trends can be noted, for instance the typical study included in this review is relatively small, involving roughly 90 cancer patients, and 64 control subjects, with only a single paper detailing a cancer patient population with more than 200 individuals. It also is common for studies to include larger populations with cancer compared to control populations, and in some cases these numbers appear quite unbalanced. For instance, the most unbalanced study involved in this review utilized 182 cancer patients compared to only 18 control subjects (20), while the second most had 122 cancer patients compared to 18 controls (21). In the more extreme cases, this study design may have negative implications for the statistical strength of these studies, decreasing their generalizability, and increasing the risk of bias influencing the conclusions of these papers. Moreover, most studies utilized hospitalized subjects with well-confirmed cancer diagnoses and at advanced stages, a consequence of which potentially being that their results may not necessarily translate into populations with earlier stage cancers, as the impact of cancer on the metabolome can evolve significantly throughout different stages of the disease (22).

**Table 3:** Sample sizes and published subject descriptions for urinary cancer voc studies published from 2020 to 2023.

Patient Group 1	Patient Group 2	Patient Group 3	Controls	Diagnostic Method	Ref
Animal Olfaction					
30 patients undergoing surgery for esophageal cancer, average age 72			N/A	nematode	(62)
40 canine cancer patients with lymphoma, mast cell tumors, melanoma or hemangiosarcomas			16 canine patients without confirmed cancer diagnosis	nematode	(63)
21 male (prostate cancer) confirmed patients, average age 65.2 ± 4.49	19 male patients with benign (prostate cancer), average age 62.6 ± 7.26		27 male patients negative for prostate cancer, average age 65.4 ± 5.33	nematode	(28)
79 female, 86 male diagnosed (lung cancer) patients, average age 68.19			51 female, 99 male patients negative for lung cancer, average age 51.22	rat	(61)
31 male, 24 female, confirmed (lung cancer) patients, average age 63.3 ± 9.5	1 male, 5 female, patients with benign lung diseases, average age 56.6 ± 6.3		8 male, 12 female healthy controls, average age 29.2 ± 2.5	dog	(23)
40 female (breast cancer) patients, median age 57.5	142 female (non-breast malignancy) patients, median age 57		18 healthy female subjects, median age 52	dog	(20)
36 histologically confirmed (lung cancer) patients, average age 66.7 (conditioning phase)	41 histologically confirmed (lung cancer) patients, average age 65.6 (study phase)		150 non-cancer patients and healthy volunteers, average age 63.7 (Conditioning phase) 205 non-cancer patients and healthy volunteers, average age 62.4 (study phase)	dog	(8)
12 male, Gleason 9-grade (prostate cancer) patients, median age 65.5			38 male, biopsy-negative patients, median age 58.5	dog	(29)
Mass spectrometry only					
26 (prostate cancer) patients, average age 66.95 ± 9.14			30 healthy subjects with no known pathologies, average age 46.21 ± 11.58	GC-MS	(22)
80 female biopsy-confirmed, untreated (breast cancer) patients, average age 49			88 healthy, female subjects, average age 42.7	GC-MS	(14)
70 female, 74 male terminal (lung cancer) patients, median age 70.5 (acid dataset)	53 female, 63 male terminal (lung cancer) patients, median age 70 (alkali dataset)		N/A	GC-MS	(53)
10 male C57BL/six mice which received transgenic prostate cancer cells in tibia	68 male patients with indolent prostate cancer	27 male patients with aggressive prostate cancer	67 suspected (prostate cancer) patients with negative biopsies	GC-MS	(47)
10 female, 30 male, histopathologically confirmed (bladder cancer) patients, average age 59.9			18 female, 39 male, healthy controls, average age 55.8	GC-MS	(42)
14 male, 7 female patients with new (urothelial bladder cancer) diagnosis, median age 67	54 male, 21 female patients with recurrent urothelial bladder cancer, median age 73	62 male, 22 female patients with non-recurrent urothelial bladder cancer, median age 69	72 male, 53 female patients with non-cancer hematuria	GC-MS	(45)
20 female BALB/c mice injected with mammary (breast cancer) tumor cells			20 female BALB/c mice prior to injection with mammary tumor cells	GC-MS	(49)
14 female, 39 male (urothelial bladder cancer) patients of various stages, median age 68.9 ± 10.6			16 female, 40 male, cancer-free controls, median age 51.9 ± 5.2	GC-MS	(51)
20 female BALB/c mice injected with mammary (breast cancer) tumors			20 female BALB/c mice prior to injection with mammary tumors	GC-MS	(15)
20 male (prostate cancer), patients, average age 67 ± 8.1	20 male (bladder cancer) patients, average age 69 ± 8.6	20 male (renal cell adenocarcinoma) patients, average age 71 ± 7.7	20 male, cancer-free controls, average age 58 ± 2.8	GC-MS	(43)
24 female (breast cancer) patients, average age 52			27 healthy subjects, average age 44	TOF-MS	(48)
43 histologically diagnosed (prostate cancer) patients, average age 68.3 ± 6	23 histologically diagnosed (prostate cancer) patients, average age 66 ± 6.8		87 non-cancer patients, average age 65 ± 11.9 and 65.5 ± 7.9 (two subsets)	TOF-MS	(58)
eNose only					
132 male (prostate cancer) patients of various grades			19 female, 41 male healthy volunteers with negative family history of prostate cancer	eNose	(16)
110 (renal cell adenocarcinoma) patients with confirmed neoplasms, average age 64.53 ± 11.72			142 ambulatory patients, no previous evidence of neoplasms, average age 63.05 ± 14.27	eNose	(9)
5 female, 26 male (hepatocellcular carcinoma) patients, median age 71	62 male (prostate cancer), patients, median age 71.2	5 female, 24 male (bladder cancer), patients, median age 74.2	7 female, 11 male non-cancer patients referred for investigation of abdominal symptoms	eNose	(21)
Combination/Others					
22 male, 23 female, (pancreatic ductal adenocarcinoma) patients, average age 64.1 ± 10.7	28 male, 17 female patients with chronic pancreatitis, average age 52.9 ± 12.2		15 male, 18 female healthy controls, average age 49.9 ± 7.8	GC-MS & GC-IMS	(27)
12 male, 3 female (bladder cancer) patients, average age 70.0	55 male (prostate cancer) patients, average age 71.9		24 male, 12 female, non-cancer patients, average age 62.5	GC-MS & GC-IMS	(57)
39 female (breast cancer) patients, average age 54.9 ± 12.0			51 female subjects, average age 29.7 ± 15.8	eNose and GC-MS	(24)
12 female (cervical cancer) patients, average age 47.4 ± 2.4	5 female patients with cervical intraepithelial neoplasms, average age 35.2 ± 1.6		12 healthy female controls, average age 35.3 ± 1.7	eNose and GC-MS	(44)
26 pathologically confirmed (gastric cancer) patients	101 pathologically confirmed (renal cell adenocarcinoma) patients	26 pathologically confirmed (non-small cell lung cancer) patients	130 non-cancer subjects exhibiting gastric symptoms	eNose and GC-MS	(60)
52 patients with confirmed urinary bladder cancer	7 patients with fresh hematuria	5 patients with other cancer types	21 “normal” volunteers	Fluorometric sensor array	(38)
4 male, 1 female patient with confirmed bladder cancer, average age 61.8			4 male, 1 female patients from Urology Outpatient Department, average age 60.8	Fluorometric sensor array	(37)

N/A, Not applicable.

More concerningly however, is the tendency for authors to select cancer and non-cancer test groups with a substantial difference in age, with many studies detailing a cancerous population ten or more years senior compared to their control group. Three studies in particular, detail sample and control populations with more than two decades separating the average ages of the sampling groups (22–24). While these differences should be considered a major confounding factor for these studies, it also does make sense that these studies likely face difficulties in recruiting younger cancer patients, given that the risk of developing cancers generally increases with age (25). Overall, however while some risk factors exist in the sampling strategies of the analyzed studies, many of these issues are simply a consequence of these studies being preliminary in nature, and presumably larger samples sizes of a few thousand individuals will eventually be conducted in the future to validate these initial findings (26).

Finally, in terms of the types of control populations used, roughly one half of studies employed subjects described as either ‘healthy or normal’, 45% specified that they utilized subjects with confirmed negative cancer diagnoses, 26% described their controls as ‘patients’, typically drawn from the non-cancerous patient population of a hospital or clinic, while a mere 10% of studies specified that they utilized non-cancerous subjects experiencing symptoms relevant to a particular type of cancer. This last category should be considered particularly important when comparing results, as a mature VOC-based diagnostic test would presumably be most useful if it could be applied on patients experiencing newly acquired or minor symptoms characteristic of a particular type of cancer, but which had yet to receive more thorough testing. For example, in one study, the researchers choose to include two control sets, one of which was comprised of healthy subjects, while the other were experiencing another, non-cancer disease (27). On the other hand, two groups sought to address this issue by using sets of patients recommended to undergo a conventional cancer test, presumably due to their experience of cancer-related symptoms (28, 29). Those patients which subsequently tested negative for cancer were then selected as the control population.

### 2.3 Study design and diagnostic accuracies

The reviewed studies explored a range of experimental strategies, including differences in detection platforms and sample handling techniques, for the detection of VOCs related to cancer. Some of these methodological differences could be expected to considerably impact on the accuracy and consistency a developed cancer detection model can achieve. **Table 4** outlines the VOC detection platforms, sample handling techniques, and diagnostic accuracies employed by the reviewed studies.

**Table 4:** Detection platforms for urinary cancer vocs and diagnostic strengths.

Platform Used	VOC Extraction Method	Storage Conditions	Sensitivity	Specificity	Ref
Animal (dog)	Direct exposure	Storage at -20°C	low response to urine samples - Not reported	low response to urine samples - Not reported	(23)
Animal (dog)	Direct exposure	Storage at -20°C for “a few days”	100%^1^	100%^1^	(20)
Animal (dog)	Direct exposure	Storage at -80°C	85%^1^	95%^1^	(8)
Animal (dog)	Direct exposure	Storage at -80°C	71.4% (dog #1), 71.4% (dog #2)	76.2% (dog 1), 76% (dog)	(29)
Animal (dog)	Direct exposure	Storage at -20°C for 2-4 weeks	65.7-90% (run #1, two lung cancer types), 60-80% (run #2, two lung cancer types)	25% (run #1, two lung cancer types), 29.2% (run #2, two lung cancer types)	(10)
Animal (dog)	Direct exposure	Storage at -20°C	100% (dog #1), 98.6% (dog #2)	98.7% (dog #1), 97.6% (dog #2)	(67)
Animal (rats)	Direct exposure, samples heated to 60°C	Storage at -20°C	93%^1^	91%^1^	(61)
Animal (nematode)	Direct exposure	Storage at -20°C	100% (untreated *vs*. successfully treated cancer)	63% (untreated *vs*. successfully treated cancer)	(62)
Animal (nematode)	Direct exposure	Storage at -20°C	88% (run #1), 85% (run #2) (all cancer types *vs*. controls)	93% (run #1), 90% (run #2) (all cancer types *vs*. controls)	(63)
Animal (nematode)	Direct exposure	Storage at -80°C	76%^1^	67%^1^	(28)
eNose	Direct exposure, samples heated to 37°C	Immediately run	89.4%^1^	71.8%^1^	(9)
eNose	Headspace sampling, samples heated to 37°C for 50 minutes	Storage at -18°C	82%^1^	87%^1^	(16)
eNose	SPME extraction of VOCs, samples heated to 23°C for 5 minutes	Storage at -80°C	100% (hepatocellcular carcinoma *vs*. all other groups), 100% (prostate cancer *vs*. all other groups), 86% (bladder cancer *vs*. all other groups)	100 (hepatocellcular carcinoma *vs*. all other groups), 96% (prostate cancer *vs*. all other groups), 97% (bladder cancer *vs*. all other groups)	(21)
eNose and GC-MS	(eNose) Thermal desorption tube for volatile extraction, samples heated to 270°C (GC-MS) Headspace sampling, samples heated to 70°C for 50 minutes	Storage at -80°C	78%^1^ (GC-MS model), 95.8%^1^ (2 runs, sensor model), 83.5-91.1%^1^ (2 runs, sensor model #2)	70%^1^ (GC-MS model), 76.7-84.6%^1^ (2 runs, sensor model), 96.15-93.3%^1^(2 runs, sensor model #2)	(60)
eNose and GC-MS	(eNose) Headspace sampling, samples heated to 20°C for 40min (GC-MS) Headspace sampling, samples heated to 150°C for 20 minutes	Storage at 4°C for less than 48 hours	100%^1^ (GC-MS model), 100%^1^ (eNose model)	85.071%^1^ (GC-MS model), 50%^1^ (eNose model)	(24)
eNose and GC-MS	(eNose) Headspace sampling (GC-MS) SPME extraction of volatiles, samples heated to 65°C for 80 minutes, SPME fiber exposed for 40 minutes	Storage at -80°C for less than 7 days	91.6%^1^	100%^1^	(44)
GC-MS	SPME extraction of volatiles, samples heated to 50°C for 60 minutes	Storage at -20°C	100%^1^	83.3%^1^	(22)
GC-MS	SPME extraction of volatiles, samples heated to 60°C for 30 minutes	Storage at -20°C or -80°C	N/A	N/A	(53)
GC-MS	SPME extraction of volatiles, samples heated to 50°C for 60 minutes, guanidine hydrochloride added	Storage at -80°C,	75%^1^ (6 VOC model), 78% (aggressive, *vs*. non-aggressive cancer, 7 VOC model)	69%^1^ (6 VOC model), 85% (aggressive, *vs*. non-aggressive cancer, 7 VOC model)	(47)
GC-MS	SPME extraction of volatiles, samples heated to 45°C for 75 minutes, SPME fiber exposed for 45 minutes	Storage at -20°C	N/A	N/A	(42)
GC-MS	SPME extraction of volatiles, samples heated to 60°C for 30 minutes	Storage at -20°C or -80°C	71%^1^ (8 VOC model), 71%^1^ (6 VOC model)	72%^1^ (8 VOC model), 80%^1^ (6 VOC model)	(45)
GC-MS	SPME extraction of volatiles, samples heated to 60°C for 60 minutes, SPME fiber exposed for 30 minutes, guanidine hydrochloride added	Storage at -80°C	91% (cancer week 1-3 *vs*. controls), 100% (cancer week 3 *vs*. controls)	100% (cancer week 1-3 *vs*. controls), 100% (cancer week 3 *vs*. controls)	(49)
GC-MS	SPME extraction of volatiles, samples heated to 44°C for 41 minutes, SPME fiber exposed for 30 minutes, part of samples derivatized with O-(2,3,4,5,6-pentafluorobenzyl)hydroxylamine hydrochloride (PFBHA)	Storage at -80°C,	65% (early-stage cancer *vs*. controls), 94% (middle-stage cancer *vs*. controls), 60% (late-stage cancer *vs*. controls)	84% (early-stage cancer *vs*. controls), 80% (middle-stage cancer *vs*. controls), 91% (late-stage cancer *vs*. controls)	(51)
GC-MS	SPME extraction of volatiles, samples heated to 60°C for 60 minutes, SPME fiber exposed for 30 minutes, guanidine hydrochloride added	Storage at -80°C	98% sensitivity (all cancer *vs*. controls, 44 VOC model), 97% (cancer week 1 and 3 *vs*. controls, 44 VOC model), 98% (all cancer *vs*. controls, 5 VOC model), 100% (all cancer *vs*. controls, refined model), 100% (cancer week 1 *vs*. controls, refined model)	95% sensitivity (all cancer *vs*. controls, 44 VOC model), 90% (cancer week 1 and 3 *vs*. controls, 44 VOC model), 95% (all cancer *vs*. controls, 5 VOC model), 95% (all cancer *vs*. controls, refined model), 92% (cancer week 1 *vs*. controls, refined model)	(15)
GC-MS	SPME extraction of volatiles, samples heated to 62°C for 57 minutes, SPME fiber exposed for 51 minutes, part of samples derivatized with O-(2,3,4,5,6-pentafluorobenzyl)hydroxylamine hydrochloride (PFBHA)	Storage at -80°C	78% (prostate cancer *vs*. renal cancer), 72% (prostate cancer *vs*. controls), 76% (prostate cancer *vs*. renal cancer and controls)	100% (prostate cancer *vs*. renal cancer), 100% (prostate cancer *vs*. controls), 97% (prostate cancer *vs*. renal cancer and controls)	(43)
GC-MS	SPME extraction of volatiles, samples heated to 60°C for 60 minutes, SPME fiber exposed for 30 minutes, guanidine hydrochloride added	Storage at -80°C	N/A	N/A	(65)
GC-MS	SPME extraction of volatiles, samples heated to 62°C for 57 minutes, SPME fiber exposed for 51 minutes, part of samples derivatized with O-(2,3,4,5,6-pentafluorobenzyl)hydroxylamine hydrochloride (PFBHA)	Storage at -80°C	78%^1^ (model #1), 100%^1^ (model #2), 89%^1^ (6 VOC model)	94%^1^ (model #1), 100%^1^ (model #2), 83%^1^ (6 VOC model)	(50)
GC-MS	Thermal desorption tube for volatile extraction, sample heated to 45°C, increasing to 300°C	Storage at -80°C	96%^1^ (validation set), 87%^1^ (evaluation set)	80%^1^ (validation set), 77%^1^ (evaluation set)	(41)
GC-MS	SPME extraction of volatiles, samples heated to 60°C for 60 minutes, SPME fiber exposed for 30 minutes	Storage at -20°C	85-95%^1^ (8 different single VOC models)	70-100%^1^ (8 different single VOC models)	(46)
GC-MS	SPME extraction of volatiles, samples heated to 45°C for 60 minutes, SPME fiber exposed for 50 minutes, sample filtered through 30kDa, 10kDa and 3kDa membranes to obtain different fractions	Storage at -80°C	63-75%^1^ (mean values, 4 different models)	35-53%^1^ (mean values, 4 different models)	(55)
GC-MS	SPME extraction of volatiles, samples heated to 60°C for 60 minutes, SPME fiber exposed for 30 minutes	Storage at -80°C	76.3%^1^ (training set), 76.0%^1^ (validation set)	85.4%^1^ (training set), 92.3%^1^ (validation set)	(14)
GC-MS and GC-IMS	(GC-IMS) Headspace sampling, samples heated to 40°C for 10 minutes (GC-MS) Thermal desorption tube for volatile extraction, samples heated to 40°C for 10 minutes	Storage at -80°C	52% (cancer *vs* all), 72% (cancer *vs*. healthy), 38% (cancer *vs*. pancreatitis)	96% (cancer *vs*. all), 96% (cancer *vs*. healthy), 88% (cancer *vs*. pancreatis)	(27)
GC-MS and GC-IMS	(GC-IMS) Headspace sampling, samples heated to 40°C for 10 minutes (GC-MS) Thermal desorption tube for volatile extraction, samples heated to 40°C for 20 minutes	Storage at -80°C	87% (GC-IMS, bladder cancer *vs*. control), 76% (GC-IMS, prostate cancer *vs*. control), 27% (GC-TOF-MS, bladder cancer *vs*. controls), 78% (GC-TOF-MS, prostate cancer *vs*. controls)	92% (GC-IMS, bladder cancer *vs*. controls), 88% (GC-IMS, prostate cancer *vs*. controls), 94% (GC-TOF-MS, bladder cancer *vs*. controls), 88% (GC-TOF-MS, prostate cancer *vs*. controls)	(57)
TOF-MS	Headspace sampling, samples heated to 50°C for 4 minutes	Storage at -80°C	92.6%^1^ (3 VOC model)	91.2%^1^ (3 VOC model)	(48)
HS-PTV-MS^2^	Headspace sampling, samples heated from 35°C to 250°C	Storage at -20°C	100%^1^ (3 different models)	91-100%^1^ (3 different models)	(66)
HS-PTV-MS	Headspace sampling, samples heated from 35°C to 250°C	Storage at -20°C	40-100%^1^ (4 different single VOC models)	100%^1^ (4 different single VOC models)	(59)
GC-FID	Headspace sampling, samples heated to 95°C for 20 min	Storage at -80°C	84.8% (head and neck cancer *vs*. controls), 66.1% (gastric cancer *vs*. controls)	82.3% (head and neck cancer *vs*. controls), 77% (gastric cancer *vs*. controls)	(11)
GC-IMS	Headspace sampling, samples heated to 80°C for 5 minutes	Storage at -20°C or -80°C	100% (colorectal cancer *vs*. control), 48% (colorectal cancer and adenomas *vs*. controls	92% (colorectal cancer *vs*. control), 89% (colorectal cancer and adenomas *vs*. controls	(64)
GC-IMS	Headspace sampling, samples heated to 100°C for 5 minutes	Storage at -80°C	52.2-87%^1^ (different models)	91.3-95.7%^1^ (different models)	(58)
GC-IMS	Headspace sampling, sample heated to 40°C for 30 seconds	Storage at -70°C	79%^1^	79%^1^	(18)
GC-IMS	Headspace sampling, sample heated to 40°C for 5 minutes	Storage at -80°C	91%^1^, 91% (early-stage cancer *vs*. controls), 82% (early-stage cancer *vs*. late-stage cancer)	83%^1^, 78% (early-stage cancer *vs*. controls), 89% (early-stage cancer *vs*. late-stage cancer)	(19)
Fluorometric sensor array	Headspace sampling, sample heated to 37°C for 15 minutes	Not stated	87%^1^	86%^1^	(38)
Fluorometric sensor array	Headspace sampling, sample heated to 37°C for 5 minutes	Storage at -20°C	78%^1^	93%^1^	(37)
LC-MS, NMR	Incomplete	Incomplete	Incomplete	Incomplete	(31)

^1^Cancer *vs*. controls/health subjects.

^2^Headspace positive temperature vaporization mass spectrometry. N/A, Not applicable.

#### 2.3.1 VOC detection platforms

Regarding detection platforms, mass spectrometry (MS), used in 24 of the reviewed studies, was by far the most popular, and was frequently combined with gas chromatography or used in conjunction with other methods. MS functions by ionizing compounds and then separating the resulting fragments according to their mass to charge ratio, as a result such techniques can identify thousands of individual VOCs within sample, and even provide some information regarding their concentration (30). Nuclear magnetic resonance spectroscopy (NMR) is another platform capable of identifying specific VOCs and was utilized in a single paper in this review. This method employs strong magnetic fields to probe the local magnetic fields of samples in order analyze molecular structures. NMR techniques in turn have the advantage of relatively simple sample preparation, allowing for non-destructive analyses of compounds, and enabling the absolute identification of individual VOC concentrations. On the other hand NMR can be less sensitive compared to MS, though the development of new probes for NMR has begun to address this issue (31, 32). However, both MS and particularly NMR systems come with high initial equipment and operating costs. The fact that despite these high costs most of the studies in this review still chose to employ some variety of MS may indicate that researchers are currently more interested in identifying specific VOCs that occur in cancer patients which can be targeted in future studies. Alternatively, MS platforms may also simply be relatively available to researchers, given that they are commonly installed in universities and research institutions, where they can be leased as needed for a particular project.

Ion-mobility spectrometry (IMS), a method related to MS, was employed by six studies and instead separates ion fragments based on their mobility through a carrier gas in an electrical field. These techniques are often characterized by higher speeds than MS but may not provide as high a resolution in exchange (27). Flame ionization detection (FID) in turn is a lower cost system relative to MS and IMS, which similarly seeks to measure generated ions but instead uses a flame to burn initial compounds. Only a single study in this review employed this technique, possibly because such systems are less suitable for identifying specific VOCs when used alone (11).

However, while identifying specific VOCs associated with cancer patients is useful, it is not strictly necessary – only that cancer related VOCs, or patterns thereof can be detected. Animal olfaction operates on this principle, with animals being trained to recognize a particular smell in an initial training period, and then responding when it encounters an identical or similar smell in the future. Animal olfaction was the second major group of VOC detection platforms observed in this review, with 10 studies employing one or more animals to recognize and respond to VOC signatures from a sample, with dogs being the most popular animal followed by nematodes and then rats. Importantly, many animals can detect very low concentrations of certain VOCs – which may in some cases exceed the accuracies of MS platforms. For instance, Tert-butyl mercaptan is detectable around 0.3 parts per billion by the human nose (33), while dogs exhibit even lower levels of detection at concentration of one part per trillion (34), levels of detection comparable to those for MS platforms (35). However, these methods can yield data which may be more difficult to objectively quantify, and which may be less consistent overall due to changes in the animal’s attention, mood and physical health over time or between different animals (20, 34). Furthermore, the animals in question must be trained to recognize and respond to VOCs of interest correctly, a process that takes time, and which is not guaranteed to ultimately succeed (23). Finally, the use of vertebrate and “higher animals” in scientific research necessitates the raising of important questions and considerations related to the ethical treatment of these creatures, potentially complicating their use as diagnostic tools (36).

Sensor-based technologies, often described as ‘electronic noses’ (eNose), constituted the third major group of VOC detection platforms. As their name implies, these technologies seek to mimic animal olfaction using individual, or arrays of sensors, that measure the physical or chemical properties of a gas, such as electrical conductivity or resistance (16, 21). Notably, eNose technologies address some of the potential limitations of animal olfaction, as eNose responses can be expected to be fully consistent between measurements so long as the equipment is not degraded, and responses can be more easily quantified for analytical purposes.

Finally, two studies in this review employed light-based fluorescence sensor arrays which very generally used the detection of specific wavelengths of light to detect and quantify specific VOC compounds in a particular sample (37, 38). These methods are relatively inexpensive compared to MS-based methods and can provide a way to semi-quantify compatible VOCs. However, these methods may also be somewhat more limited in terms of only being suitable for specific VOC’s compatible with the specific method used, as well as potentially requiring the addition of reagents to facilitate measurement, potentially explaining their relative rarity in this review (25, 26).

#### 2.3.2 Sample handing techniques

Aside from detection platforms, sample handling techniques should also be considered important when developing a VOC-based model of cancer detection. For instance, several detection platforms, such as MS, typically require concentrating a sample prior to analysis (39). One technique used by many of the reviewed studies to achieve this concentration is known as headspace sampling, wherein VOCs are collected from the gas phase above a sample in a sealed container. This method in turn is also frequently paired with a technique known as solid phase microextraction (SPME), which uses a small fiber coated with a sorbent material to absorb the VOCs in the headspace of a sample, before being transferred to an analytical system where it can then be heated to release collected compounds. Importantly SPME has the advantage of being low-cost, simple, and not requiring solvents or highly specialized equipment (40). As a result, it was unsurprising that many of the studies employing GC-MS utilized SPME alongside it. However, it should be stated that SPME does impose an upper limit on the concentration of VOCs that can be collected at one time on the fiber, and moreover different compounds may attach to the fiber at different rates – causing there to be no guarantee that SPME will extract a representative set of sample VOCs (21, 22, 40).

Thermal desorption was another method used for concentrating sample VOCs. In these techniques samples are heated in an airtight tube, and a carrier gas transports VOCs to the analytical instrument. This method addresses some of the limitations of SPME, as thermal desorption can allow for the collection of very low concentration VOCs, as well as allowing for the capture of semi-volatile compounds, and providing quantitative or near-quantitative desorption of VOCs (39, 41). However, this method does require specialized equipment and can be somewhat more complex in comparison to SPME, potentially explaining its relative rarity in the reviewed studies. While both SPME and thermal desorption enhance VOC concentration, there were scenarios in the reviewed literature where direct exposure of the samples evidentially was sufficient, as was the case for many of the animal-based detection systems in this review. In these cases, samples are simply placed near a detector for the VOCs to passively diffuse into. This allows for rapid and straightforward collection of VOCs but may suffer from reproducibility issues due to changing environmental factors such as temperature and humidity variations (8).

#### 2.3.3 Sample collection techniques

Many authors did not include a high level of detail on the specific methods used for urine sample collection. This is understandable, as urine testing can be as simple as providing a container to a patient and requesting that they fill part of it. However, there were exceptions to this trend. For instance, some groups specified that only early morning urine specifically was collected (16, 42, 43). This type of sampling is preferable in many instances as overnight the body is typically in a state of low hydration, leading to more concentrated urine and therefore potentially resulting in more and more concentrated VOCs in the sample. Moreover, since diet, hydration and physical activity levels throughout the day can influence urine composition, collecting early morning urine specifically may produce a more consistent baseline between different individual. Some authors further enhanced this approach by also ensuring that the collection of early-morning urine samples was preceded by an overnight fasting period (14, 44).

Other studies specified that mid-stream urine was collected (27, 42). This technique can help to reduce contamination from debris such as cells, or bacteria in the urethra, enhancing sample reliability. In a related approach one group obtained samples from catheterized patients, and then divided their urine samples into different fractions depending on time of excretion (16). While this approach could offer further control regarding contamination, it is likely infeasible for most patients due to its highly invasive nature.

#### 2.3.4 Sample storage techniques

Storage conditions of urine samples was another component of studies that was found to vary between studies. In particular, some researchers advocated for -80°C storage of urine samples as being necessary to preserve VOC signatures (45), while others contended that -20°C was sufficient (46), one group even opted not to freeze their samples at all, storing their samples at 4°C, and another group opted to avoid storage altogether by immediately running their samples following collection (9). Several studies also experimented with modifying their urine samples to enhance VOC release. For instance, many of the reviewed methods heated the urine samples when analyzing them, with temperatures ranging from 37°C to well beyond the boiling point of water. This process increases the kinetic energy of VOCs leading to faster release of VOCs from a sample and can result in a more concentrated headspace above the sample. However certain VOCs may also degrade or undergo chemical transformation, potentially altering their composition or reducing the number of detectable VOCs. Similarly, many studies sought to modify the pH or salinity of their samples, the former of which may stabilize or destabilize certain VOCs, while increasing salinity can alter the solubility of some compounds – driving or pulling them away from the headspace (45, 47, 48). In a similar vein, guanidine hydrochloride was used by several studies in order to denature proteins and facilitate VOC release (47, 49), while O-(2,3,4,5,6-pentafluorobenzyl)hydroxylamine hydrochloride (PFBHA) (43, 50, 51) has been used to derivatize carbonyl compounds and thereby make them more detectable in subsequent analyses.

#### 2.3.5 Diagnostic accuracy

The final step in developing a VOC-based cancer detection model is evaluating its diagnostic accuracy. To do this, authors usually seek to describe two statistical properties of the test, sensitivity, and specificity. The former property refers to the ability of a test to correctly identify individuals who have the disease in question, and as such can also be described as the “true-positive” rate. Specificity in turn is used to describe the ability of a test to correctly identify individuals who do not have the disease being tested for, or the true negative rate. Both sensitivity and specificity are usually described as a percentage of the surveyed population. As perfect sensitivity and specificity is usually incredibly difficult to achieve, a general rule of thumb is that if the two accuracy scores sum together for more than 150%, then a given test possesses at least some diagnostic value (52).

Interestingly, according to **Table 4**, most of the studies in this review achieved this benchmark, indicating that the models developed by these studies could plausibly be used to diagnose cancer based on VOC signatures. Moreover, no obvious trends exist between the diagnostic power of all the different platforms analyzed, though eNose-based systems may be seen to have a slightly higher accuracy overall, while animal and GC-MS based systems appear to display larger variances in their effectiveness when comparing study to study. In addition, the significance of storage was also unclear with respect to these values as samples stored at -80°C, generally did not appear to result in increased accuracy of the produced model when compared to -20°C storage, and even the one study which used 4°C still achieved a combined sensitivity and specificity of 150% or higher with their models. Moreover, the value of immediate analyses is also somewhat unclear as one group which opted to avoid long term storage still achieved a relatively unremarkable combined sensitivity and specificity of 169% (9). It may be notable that one study, which required patients to undergo an 8-hour fast followed by early morning urine collection obtained a relatively high combined sensitivity and specificity value of 192% (44), though another study following similar protocols only achieved a combined value of 161.7% (14). Differences in VOC extraction methods similarly did not appear to have a clear effect on model accuracies, though it is potentially noteworthy that two studies which used guanidine hydrochloride both achieved combined sensitivity and specificities approaching 200% (47, 49). However, it is important to note that it may be difficult to draw any concrete conclusions from a direction comparison of the accuracy scores of the various articles, given that different studies had very different sets of goals and operated using diverse sets of samples.

### 2.4 Volatile urinary metabolites of cancer

**Figure 1:**
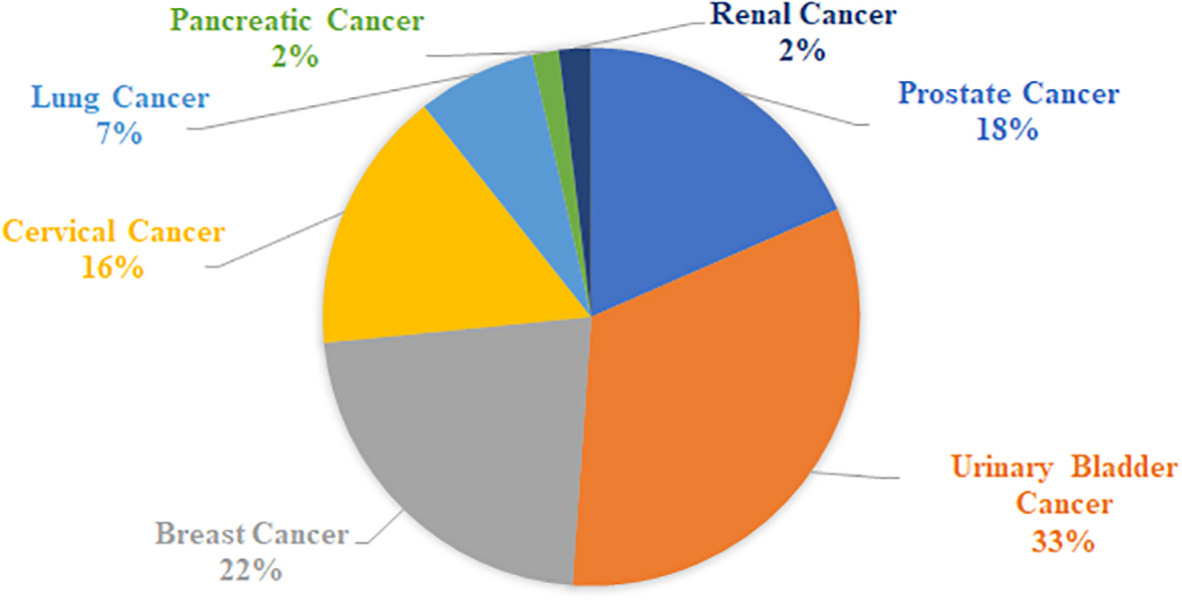
Relative proportion of cancer types in **Supplementary Table S1**.

**Figure 2:**
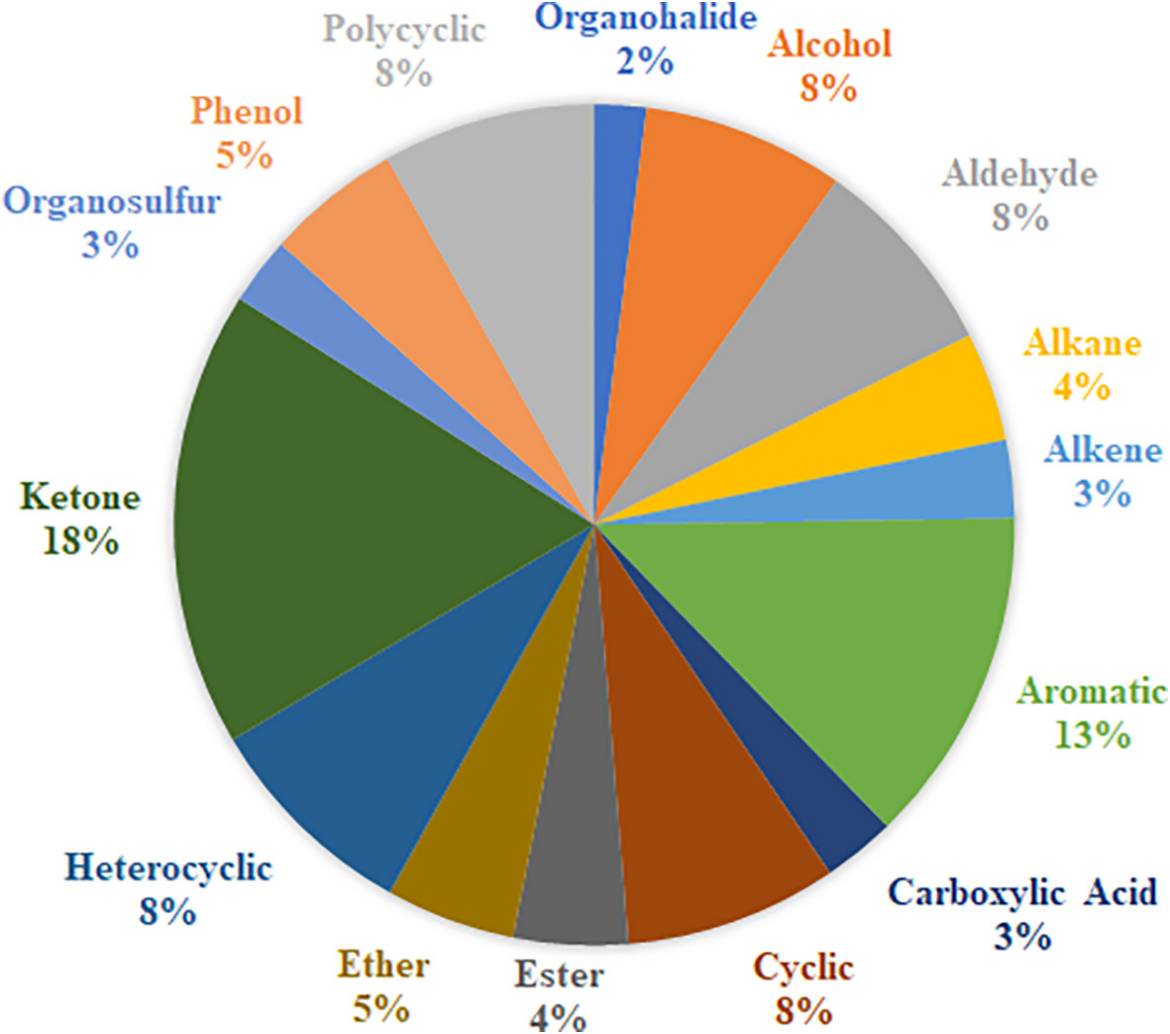
Common functional groups of volatile compounds identified with cancer.

As part of this review, a table listing specific VOCs significantly identified with cancer by the reviewed authors was compiled in **Supplementary Table S1**, which includes 329 separate entries and 252 unique chemical compounds. Within **Supplementary Table S1**, the relative amount of VOC entries per cancer type was also determined and further summarized into **Figure 1**. Notably the most common associations were with urinary bladder cancer (33% of database), breast cancer (22% of database), prostate cancer (18%) and cervical cancer (16%), while entries related to lung cancer, renal cancer or pancreatic cancers together occupied only 11% of the database, possibly indicating that these latter cancers may produce fewer or less useful characteristic urinary volatiles, or else the research for these types is still simply at a less advanced stage. Following this, one or more descriptors were then assigned to each of these compounds included in **Supplementary Table S1** based on their molecular structure – a summary of which can be viewed in **Figure 2**. From this figure it is apparent that the most common chemical structures included in this list are ketones (15%), aromatic compounds (13%), alcohols, aldehydes, cyclic, and polycyclic compounds (~8% each), and phenols and ethers (~5% each). From **Supplementary Table S1**, every compound identified by two or more distinct studies, 47 chemical species in total, were then refined into **Table 5** for further discussion.

**Table 5:** List of voc biomarkers of identified or utilized in at least two separate papers.

Compound Name	Formula	Molecular Weight	Cancer Type	Identification Technique	Concentration / Change in Signal Magnitude	Ref
propan-2-one	C_3_H_6_O	58.0790	Lung	MS	Increased 1.382-fold	(53)
			Bladder	MS		(45)
pyrrole	C_4_H_5_N	67.090	Lung	MS	Not significantly different	(59)
			Prostate	IMS		(57)
butan-2-one	C_4_H_8_O	72.1057	Breast	MS		(49)
			Lung	MS	Significantly increased	(59)
			Bladder	MS	Downregulated	(51)
			Breast	MS		(47)
			Breast	TOF-MS	Increased 1.58-1.92-fold	(48)
pentanal	C_5_H_10_O	86.1324	Prostate	MS	Increased	(46)
			Prostate	Calibration standard		(28)
pentan-2-one	C_5_H_10_O	86.1324	Pancreatic	IMS		(27)
			Lung	TOF-MS	Increased	(55)
			Bladder	TOF-MS	Increased 2.3-fold	(42)
			Lung	MS	Significantly increased	(59)
			Bladder	IMS		(57)
			Breast	TOF-MS	Decreased 0.38-0.56-fold	(48)
toluene	C_7_H_8_	92.1397	Breast	MS		(49)
			Prostate	IMS		(57)
phenol	C_6_H_6_O	94.1120	Bladder	MS	Increased 1.991-fold	(45)
			Prostate	MS		(22)
			Prostate	IMS		(57)
disulfide, dimethyl	C_2_H_6_S_2_	94.1990	Bladder	TOF-MS	Decreased 0.2-fold	(42)
			Prostate	IMS		(57)
cyclohexanone	C_6_H_10_O	98.1434	Lung	TOF-MS	Increased	(55)
			Bladder	TOF-MS	Increased 20.4-fold	(42)
hexanal	C_6_H_12_O	100.1591	Bladder	Calibration standard		(38)
			Prostate	MS		(43)
			Bladder	MS	Downregulated	(51)
			Bladder	Calibration standard		(37)
hexan-2-one	C_6_H_12_O	100.1591	Breast	MS		(49)
			Breast	TOF-MS	Decreased 0.56-fold	(48)
2-butanone, 3,3-dimethyl-	C_6_H_12_O	100.1591	Breast	MS		(49)
			Breast	MS		(47)
benzaldehyde	C_7_H_6_O	106.1230	Breast	MS		(49)
			Bladder	MS		(45)
ethylbenzene	C_8_H_10_	106.1664	Bladder	Calibration standard		(38)
			Prostate, Bladder & Renal	MS		(43)
			Bladder	Calibration standard		(37)
4-methylpent-3-enoic acid	C_6_H_10_O_2_	114.1424	Bladder	MS	Increased 1.457-fold	(45)
			Bladder	TOF-MS	Found only in cancer group	(42)
heptanal	C_7_H_14_O	114.1859	Bladder	TOF-MS	Found only in cancer group	(42)
			Bladder	IMS		(57)
heptan-(2 or 4)-one	C_7_H_14_O	114.1859	Bladder	TOF-MS	Decreased 0.8-fold	(42)
			Bladder	MS	Downregulated	(51)
			Breast	MS		(49)
			Bladder	MS	Increased 1.177-fold	(45)
			Prostate, Bladder & Renal	MS		(43)
			Lung	MS	Significantly increased	(59)
			Bladder	IMS		(57)
2-methoxyphenol	C_7_H_8_O_2_	124.1377	Bladder	MS	Increased 1.287-fold	(45)
			Bladder	TOF-MS	Increased 27.7-fold	(42)
oct-3-en-2-one	C_8_H_14_O	126.1969	Bladder	TOF-MS	Increased 8.3-fold	(42)
			Breast/bone metastasis	MS		(65)
5-ethyl-3-methyloxolan-2-one	C_7_H_12_O_2_	128.1691	Lung	MS	Increased 2.015-fold	(53)
			Bladder	MS	Increased 1.808-fold	(45)
naphthalene	C_10_H_8_	128.1727	Breast	MS		(49)
			Bladder	IMS		(57)
octan-2-one	C_8_H_16_O	128.2124	Prostate	MS	Decreased	(46)
			Prostate	Calibration standard		(28)
2-ethylhexan-1-ol	C_8_H_18_O	130.2281	Lung	TOF-MS	Increased	(55)
			Bladder	MS	Decreased 0.413-fold	(45)
			Prostate	IMS	Downregulated	(58)
			Lung	MS	Significantly increased	(59)
			Prostate	MS	More associated with cancer	(22)
			Prostate	IMS		(57)
(2,5 or 2,4)-dimethyl benzaldehyde	C_9_H_10_O	134.1764	Bladder	TOF-MS	Increased 30.5-fold	(42)
			Prostate	MS		(43)
			Breast	MS	Downregulated	(14)
			Bladder	TOF-MS	Increased 251.5-fold	(42)
1-(5-methylthiophen-2-yl) ethenone	C_7_H_8_OS	140.2037	Breast	MS		(49)
			Breast	MS		(47)
nonanal	C_9_H_18_O	142.2391	Pancreatic	IMS		(27)
			Bladder	MS	Decreased 0.306-fold	(45)
			Bladder	IMS		(57)
nonan-2-one	C_9_H_18_O	142.2391	Breast	MS		(49)
			Breast	MS		(47)
1,2,4-trimethylcyclohexane	C_9_H_18_O	142.2391	Breast/bone metastasis	TOF-MS		(65)
			Breast	MS		(47)
2-methyl-5-(prop-1-en-2-yl) cyclohex-2-en-1-one [Carvone]	C_10_H_14_O	150.2189	Bladder	MS	Downregulated	(51)
			Prostate	MS	More associated with controls	(22)
2,6,6-trimethylcyclohexa-1,3-diene-1-carbaldehyde	C_10_H_14_O	150.2189	Breast	MS		(47)
			Bladder	TOF-MS	Increased 1.8-fold	(42)
1,3,5-undecatriene	C_11_H_18_	150.2621	Breast	MS		(49)
			Breast	MS		(47)
2,6,6-trimethyl-2-cyclohexene-1,4-dione	C_9_H_12_O_2_	152.1911	Bladder	TOF-MS	Increased 3.0-fold	(42)
			Breast	MS		(47)
2,6-dimethyloct-7-en-2-ol [Dihydromyrcenol]	C_10_H_20_O	156.2658	Prostate	MS	Decreased	(46)
			Bladder	MS		(45)
			Prostate	MS	More associated with cancer	(22)
2-hydroxy-6-(propan-2-yl) cyclohepta-2,4,6-trien-1-one	C_10_H_12_O_2_	164.2021	Breast	MS		(49)
			Breast	MS		(47)
benzene, 1-methoxy-4-(1-methylpropyl)-	C_11_H_16_O	164.2454	Breast	MS		(49)
			Breast	MS		(47)
2-ethyl-5-n-propylphenol	C_11_H_16_O	164.2454	Breast	MS		(49)
			Breast	MS		(47)
undecan-2-one	C_11_H_22_O	170.2925	Breast	MS		(49)
			Breast	MS		(47)
1,3,5-trichlorobenzene	C_6_H_3_Cl_3_	181.4485	Breast	MS		(49)
			Breast	MS		(47)
1-(2,6,6-trimethylcyclohexa-1,3-dien-1-yl)-2-buten-1-one	C_13_H_18_O	190.2831	Bladder	TOF-MS	Increased 10.4-fold	(42)
			Lung	MS	Decreased 1.685-fold	(53)
			Breast	MS		(49)
1,1,4a-trimethyl-4,5,6,7-tetrahydro-3h-naphthalene-2-one	C_13_H_20_O	192.2988	Bladder	MS	Decreased 0.388-fold	(45)
			Lung	MS	Decreased 2.715-fold	(53)
2,4-di-tert-butylphenol	C_14_H_22_O	206.3255	Breast	MS		(49)
			Breast	MS		(47)
8,8,9-trimethyl-deca-3,5-diene-2,7-dione	C_13_H_20_O_2_	208.2978	Breast	MS		(49)
			Breast	MS		(47)
3-methoxy-5-(2-methylpropyl)-2-propan-2-ylpyrazine	C_12_H_20_N_2_O	208.3018	Breast	MS		(49)
			Breast	MS		(47)
propanoic acid, 2-methyl-, 2-ethyl-3-hydroxyhexyl ester	C_12_H_24_O_3_	216.3172	Breast	MS		(49)
			Breast	MS		(47)
hexadecane	C_16_H_34_	226.4426	Bladder	IMS		(57)
			Breast	MS		(47)
3,5-di-tert-butyl-4-hydroxybenzaldehyde	C_15_H_22_O_2_	234.3355	Breast	MS		(47)
			Breast	MS		(49)
dibutyl benzene-1,2-dicarboxylate	C_16_H_22_O_4_	278.3445	Breast	MS		(49)
			Breast	MS		(47)
[2,2,4-trimethyl-3-(2-methylpropanoyloxy) pentyl] 2-methylpropanoate	C_16_H_30_O_4_	286.4072	Breast	MS		(49)
			Cervical	MS		(44)
			Breast/bone metastasis	TOF-MS		(65)

#### 2.4.1 Ketones

Unsurprisingly, this secondary list once again included many compounds with one or more ketone groups. For instance, propan-2-one or 2-propanone was identified both as a biomarker of lung cancer (53), as well as a biomarker of newly developed bladder cancer (45), with the former authors attributing its production to altered fatty acid oxidation in cancer cells. Similarly, butan-2-one was found exclusively in the lung cancer population of one study (54) and was also found to be significant marker in breast cancer patients (49). In contrast however, another group observed that this metabolite was downregulated in bladder cancer, seemingly indicating a complex relationship with different types of cancers (51). Other ketones of note include pentan-2-one, which was observed to increase in cancer patients in studies of pancreatic ductal adenocarcinoma (27), bladder cancer (42), and lung cancer (55). However, two of these authors (42, 55) went on to clarify that this metabolite was also associated with several other diseases including Crohn’s disease, inflammatory bowel disease and diabetes which may impair the usefulness of this marker in a hypothetical diagnostic method. The most cited biomarker between all studies however was heptan-(2 or 4)-one, which was identified as significant by a total of seven studies, including two where it was found downregulated (42, 51), as well as two where it was found upregulated (45, 54). Another potential explanation for this relationship was proposed by one group, in which the authors proposed that elevate levels of methyl-ketones the feces of lung cancer patients was caused by a disrupted microbiome metabolism brought on by the disease (56).

Overall, it appears to be typical for these biomarkers to show both up- and down- regulation in different cancers as reported by different authors. Some exceptions to this trend however exist within 5-ethyl-3-methyloxolan-2-one, which was found to increase roughly 2-fold in both lung cancer (53), and bladder cancer (45). Carvone, in turn was found downregulated in studies of bladder cancer (51), and prostate cancer (22), while 1,1,4a-trimethyl-4,5,6,7-tetrahydro-3h-naphthalene-2-one decreased in expression between 0.388- and 2.715-fold in studies of bladder cancer (45), and lung cancer (53). In general, the various authors did not attempt to definitively identify why these biomarkers were capable of diagnosing cancer cases, with most authors simply proposing possibly broad metabolic mechanisms which could have generated a given compound.

#### 2.4.2 Aldehydes

Various aldehydes also demonstrated consistent associations with cancer. For example, hexanal was found to be significant in four separate articles for both bladder (37, 38, 51), and prostate cancer (43). One group further went on to attribute their observed decrease in this metabolite to the oxidation of aldehyde dehydrogenase in cancer cells, or increased lipid peroxidation (51). Different conformations of dimethyl benzaldehyde were also found to be significant by several authors and in varying concentrations across cancer types, for bladder cancer (42), prostate cancer (22, 43), and breast cancer patient (14). In one of these studies, a particularly striking increase in concentration for this metabolite was observed, increasing between 30.5- and 251.1-fold for bladder cancer patients, with the authors hypothesizing that these marked differences owed to significantly altered fatty acid metabolism in cancer cells (42). However, another study observed a decrease of this metabolite in their breast cancer population, and attributed this change to modified activity of the cytochrome p450, or an impairment of the oxidation phosphorylation process in the breast cancer cells (14). Another metabolite implicated in multiple cancer studies was nonanal, for pancreatic ductal adenocarcinoma (27), and bladder cancer (45, 57). Interestingly, several different explanations were proposed regarding the nature of this biomarker, with one group speculating that it could have arisen from the reduction of the carboxy group of nonanoic acid and proposing that it may be a non-specific marker of inflammation or general illness and not cancer per se (27). Another theorized that this relationship between nonanal and cancer, which had already been observed in previous studies, could be the result of a dysregulation of an enzyme related to oxidative metabolism which itself is commonly associated with tumor initiation and promotion (57). However, this explanation may not wholly explain things, as one group instead observed a 0.306-fold decrease in nonanal (45). However, while discussing this result, these authors would later conclude that direct comparisons of this result with previous studies was difficult due to differences in sample preparation methodologies.

#### 2.4.3 Alcohols

In terms of alcohols, the most referenced chemical species was 2-ethylhexan-1-ol, however different studies have observed different relationships between this metabolite and cancer. For instance, in two separate studies focused on bladder cancer (45) and prostate cancer (58), the authors observed a significant decrease in this metabolite relative to healthy controls, with one of the groups further speculating that this VOC could play a role in cancer progression and cell apoptosis (58). On the other hand, two more studies both focusing on prostate cancer instead observed that 2-ethylhexan-1-ol was more correlated with their cancer groups and not healthy individuals (22, 57). Previously, two group also proposed that upregulation of this metabolite could be attributed directly to the metabolic activity of lung cancer cells (55, 59). Another alcohol, 2,6-dimethyloct-7-en-2-ol, or dihydromyrcenol, was identified as an important biomarker for bladder cancer, though the authors did not indicate if this was due to being up or down-regulated in patients (45). Two more studies also found this metabolite to also be a significant marker in prostate cancer patients (22, 46), with one of them further observing an upregulation of this metabolite relative to controls and speculating that this may have been caused by cancer cells utilizing this metabolite as an energy source (46).

#### 2.4.4 Other compound classes

Interestingly increased expression of phenol and 2-methoxyphenol, appeared to be a consistent feature in bladder cancer (42, 45), and was also identified as significant in prostate cancer patients (22, 57). One final metabolite of note 4-methylpent-3-enoic acid was also found to increase in bladder cancer patients in the two studies which identified it, with one group observing a 1.457-fold increase (45), while the other only observed this metabolite in the cancer group (42).

## 3 Conclusion

In this review, the number and distribution of studies related to the identification of urinary VOCs associated with different types of cancer, and their capacity to diagnose incidents of cancer was reviewed. It was noted that in the past five years, approximately six articles per year have been produced on this topic, with prostate, lung, bladder, and breast cancers being among the most extensively studied while pancreatic and colorectal cancer appears to have received less attention. It was further noted that most of the studies analyzed used human patients, with relatively small sample sizes. One major point of diversity among the studies was how control populations were selected, which may have implications for the potential usefulness of a VOC-based cancer test. A relatively large number of studies were noted to have either targeted multiple types, or grades of, cancer simultaneously and attempted to distinguish between them using characteristic VOC signatures. Regarding sample handling methods, neither VOC detection platform nor storage conditions appeared to have a clear influence on the accuracy of the generated models, however comparisons between studies with different objectives is difficult. In addition, many studies did not specify how urine samples were collected, despite there being some advantages associated with specific sampling techniques. Finally, some common VOCs associated with cancer identified in different studies were briefly highlighted. Overall, it was noted that while VOC-based testing for cancer appears to be upheld by the literature as a promising strategy for improving early cancer detection, more work may be needed before widespread adoption of these techniques will occur.

With the discovery of genomic and immunological biomarkers associated with several types of cancer and drugs to target these biomarkers, cancer is transitioning from an incurable disease to a chronic disease. VOCs may provide biomarkers for cancer screening, targets for drug development, and prognostics or pre-clinical indicators of cancer recurrence.

## Data Availability

The datasets used for this study have been included in this Review and in the **Supplementary Table**.

## Supplementary material

Supplementary Table S1 Complete list of volatile organic compounds significantly linked with cancer.
